# ICR suckling mouse model of Zika virus infection for disease modeling and drug validation

**DOI:** 10.1371/journal.pntd.0006848

**Published:** 2018-10-24

**Authors:** Yu-Hsuan Wu, Chin-Kai Tseng, Chun-Kuang Lin, Chih-Ku Wei, Jin-Ching Lee, Kung-Chia Young

**Affiliations:** 1 Institute of Basic Medical Sciences, College of Medicine, National Cheng Kung University, Tainan, Taiwan; 2 Doctoral Degree Program in Marine Biotechnology, College of Marine Sciences, National Sun Yat-Sen University, Kaohsiung, Taiwan; 3 Department of Biotechnology, College of Life Science, Kaohsiung Medical University, Kaohsiung, Taiwan; 4 Graduate Institute of Medicine, College of Medicine, Kaohsiung Medical University, Kaohsiung, Taiwan; 5 Research Center for Natural Products and Drug Development, Kaohsiung Medical University, Kaohsiung, Taiwan; 6 Department of Medical Research, Kaohsiung Medical University Hospital, Kaohsiung, Taiwan; 7 Department of Medical Laboratory Science and Biotechnology, College of Medicine, National Cheng Kung University, Tainan, Taiwan; Fundacao Oswaldo Cruz, BRAZIL

## Abstract

**Background:**

Zika virus (ZIKV) infection causes diseases ranging from acute self-limiting febrile illness to life-threatening Guillain–Barré Syndrome and other neurological disorders in adults. Cumulative evidence suggests an association between ZIKV infection and microcephaly in newborn infants. Given the host-range restrictions of the virus, a susceptible animal model infected by ZIKV must be developed for evaluation of vaccines and antivirals. In this study, we propose a convenient mouse model for analysis of neurological disorders caused by ZIKV.

**Methodology:**

Six-day-old immunocompetent ICR suckling mice were used in the experiment. Different inoculum virus concentrations, challenge routes, and challenge times were assessed. Viremic dissemination was determined in the liver, spleen, kidney, and brain through Western blot assay, plaque assay, absolute quantification real-time PCR, and histological observation. Azithromycin, a well-characterized anti-ZIKV compound, was used to evaluate the ICR suckling mouse model for antiviral testing.

**Conclusions:**

Signs of illness and neurological disease and high mortality rate were observed in mice injected with ZIKV intracerebrally (10^2^ to 10^5^) and intraperitoneally (10^3^ to 10^5^). Viremic dissemination was observed in the liver, spleen, kidney, and brain. ZIKV transmitted, rapid replicated, and induced monocyte infiltration into the brain approximately 5 to 6 days post inoculum. Azithromycin conferred protection against ZIKV-caused neurological and life-threatening diseases. The developed model of ZIKV infection and disease can be used for screening drugs against ZIKV and discovering the underlying mechanism of ZIKV pathogenesis.

## Author summary

Mosquito-borne Zika virus (ZIKV) is an emerging threat to human health worldwide. In 2007, a ZIKV outbreak was reported in the Yap Island of Micronesia and was the first outbreak outside Africa and Asia. In 2013 and 2014, another ZIKV outbreak was reported in French Polynesia, and more than 28,800 people were infected by ZIKV. In 2015, the first ZIKV outbreak in America was reported in Brazil; the Brazilian Ministry of Health reported a 20-fold increase in cases of neonatal microcephaly, which was geographically and temporally correlated with the ZIKV outbreak. Recent evidence demonstrated that ZIKV infection leads to severe syndromes, such as Guillain–Barré syndrome and microcephaly in adults and infants, respectively. Thus far, anti-ZIKV drugs and vaccines have not been developed yet. Moreover, the underlying mechanism of ZIKV pathogenesis remains unclear. In this study, we propose a small animal model of wild-type ZIKV infection and associated neurological disorders. In the animal model, ZIKV causes signs of illness and neurological disease, potentially emulating the hallmark of ZIKV infection in human. These features can be used to study the underlying mechanism of ZIKV pathogenesis. The newly developed Zika disease model provides an immunocompetent and time saving framework for development of drugs against ZIKV and ZIKV-caused diseases.

## Introduction

Mosquito-borne Zika virus (ZIKV), which belongs to the *Flavivirus* genus of the Flaviviridae family, is an emerging threat to human health worldwide [1]. The genome of ZIKV consists of a single-stranded positive sense RNA, which encodes a single polypeptide [2, 3]. The single polypeptide is processed by viral and host proteases to form mature viral proteins, including three structural proteins [core (C), pre-membrane (prM), and envelope (E)] and seven non-structural proteins (NS1, NS2A, NS2B, NS3, NS4A, NS4B, and NS5) [4]. ZIKV (strain MR766) was first isolated from sentinel rhesus monkeys in the Zika forest of Uganda in 1947 [5]. ZIKV was also isolated from *Aedes africanus* mosquito in the Zika forest. Few ZIKV infection cases were reported around African and Asian countries during 1960s–1980s, and ZIKV was neglected for years until 2007 [6]. In 2007, ZIKV infection attracted global attention due to the outbreak in the Yap Island of Micronesia, which was the first spread of ZIKV infection outside Africa and Asia [7, 8]. In 2013–2014, another ZIKV outbreak was reported in French Polynesia, and more than 28,800 people were infected by the virus [9]. Thereafter, ZIKV has spread rapidly throughout the Pacific region. Most importantly, in UK, a man who visited French Polynesia at 2014 was diagnosed with ZIKV; in this case, ZIKV RNA was detected in the semen 2 months post onset of the syndromes, which underlined the potential of sexual transmission of the virus [10]. In 2015, the first ZIKV breakout in America was reported in Brazil; the Brazilian Ministry of Health reported a 20-fold increase in cases of neonatal microcephaly, which was geographically and temporally correlated with the ZIKV outbreak [11, 12]. Most patients infected by ZIKV present mild symptoms, including moderate fever, headache, myalgia, conjunctivitis, and rash, which are similar to those of infection by other *Flavivirus*, such as dengue virus or West Nile virus [13–17]. Recent evidence demonstrated that ZIKV infection leads to severe syndromes, such as Guillain–Barré syndrome (GBS) and microcephaly in adults and infants, respectively [14–16]. GBS is an autoimmune disorder in which the immune system attacks the nervous system [18]. The key phenomenon of GBS is the infiltration of activated lymphocytes and monocytes in nerve tissues, leading to acute or subacute flaccid paralysis [13, 19]. At present, ZIKV spreads rapidly in Africa, America, and Asia Pacific [5]. No approved antivirals or vaccines are available for treatment of ZIKV infection. Therefore, a suitable animal model must be developed for investigating therapeutics or vaccines against ZIKV infection and related diseases.

Animal models for investigating ZIKV infection include nonhuman primates (NHPs) and mice [20]. Although NHPs are suitable for studying ZIKV pathogenesis and therapeutics/vaccine development, they are expensive [21]. In this regard, small animal models are a better choice for studying ZIKV [10, 21]. In the present study, we established a murine ZIKV infection animal model for evaluation of ZIKV therapeutics.

## Result

### ZIKV caused lethality in the suckling mouse model

**Fig 1:**
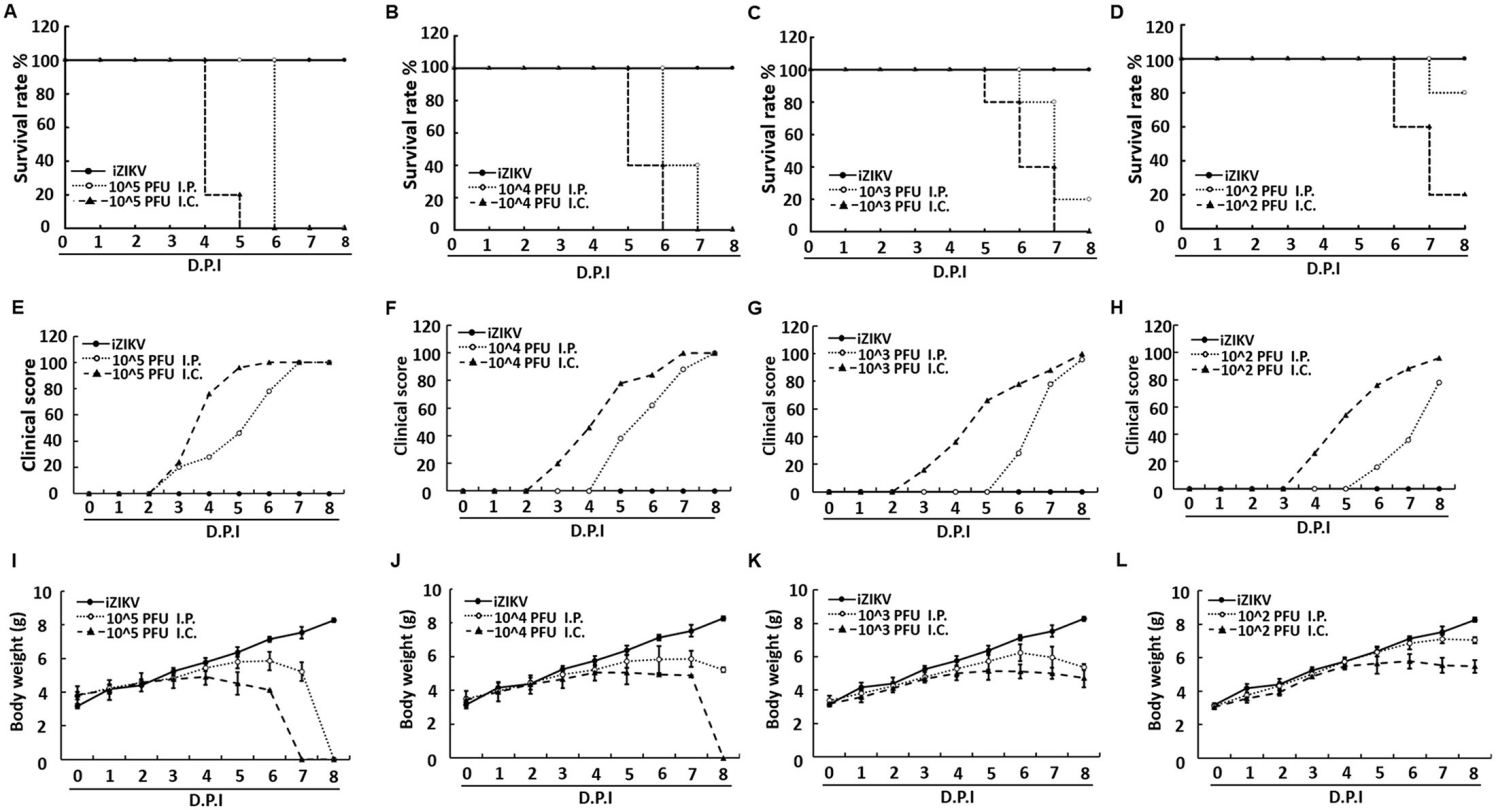
Survival rate, body weight, and clinical score of ICR suckling mice infected with ZIKV by i.p. and i.c. injection. Six-day-old ICR suckling mice received 10^2^ to 10^5^ PFU ZIKV by i.c. (n = 5) or i.p. (n = 5) injection. (A–D) Survival rate, (E–H) clinical score, and (I–L) body weight recorded every day. Mice were sacrificed at 8 dpi. Disease severity was scored as follows: 0: healthy, 1: body weight loss and ruffled fur, 2: lethargy and unsteady gait, 3: kinetic tremors and severe ataxia, 4: paralysis, and 5: death.

In initial studies, ICR suckling mice were tested for susceptibility to infection by ZIKV. Six-day-old ICR suckling mice were intracerebrally/intracranially (i.c., n = 5) and intraperitoneally (i.p., n = 5) injected with 10^2^ to 10^5^ PFU ZIKV, respectively. Mice injected with heat-inactivated ZIKV (iZIKV) were used as mock controls. Survival rate, clinical score, and body weight of ZIKV-injected mice were measured daily for 8 days. As shown in Fig 1A–1D, the i.c. injection of 10^2^ to 10^5^ PFU ZIKV resulted in 80% to 100% mortality rate at 5–7 days post infection (dpi). Meanwhile, the i.p. injection of 10^3^ to 10^5^ PFU ZIKV resulted in high mortality rate at 1 day later than the i.c. injected mice, and that of 10^2^ PFU ZIKV led to 20% mortality rate. Moreover, the i.c. injection caused death approximately 1 day earlier than the i.p. injection. Regardless of inoculation route or concentration of ZIKV, ICR suckling mice showed signs of illness and neurological disease from 2 to 5 dpi. The symptoms included body weight loss, ruffled fur, lethargy, unsteady gait, kinetic tremor, severe ataxia, and limb paralysis (Fig 1E–1H). The incidence time of clinical signs in the i.c. injection groups is approximately 2 days earlier than that in the i.p. injection groups. Interestingly, the i.p. injection groups showed rapid disease process, that is, severe paralysis or death was noted 2 to 3 days after the onset of clinical signs (Fig 1E–1H). In observation of mice morbidity, body weight loss started on 3 dpi, which is approximately the same time when clinical signs were noted (Fig 1I–1L).

### ZIKV replicated in ZIKV-infected ICR suckling mice

**Fig 2:**
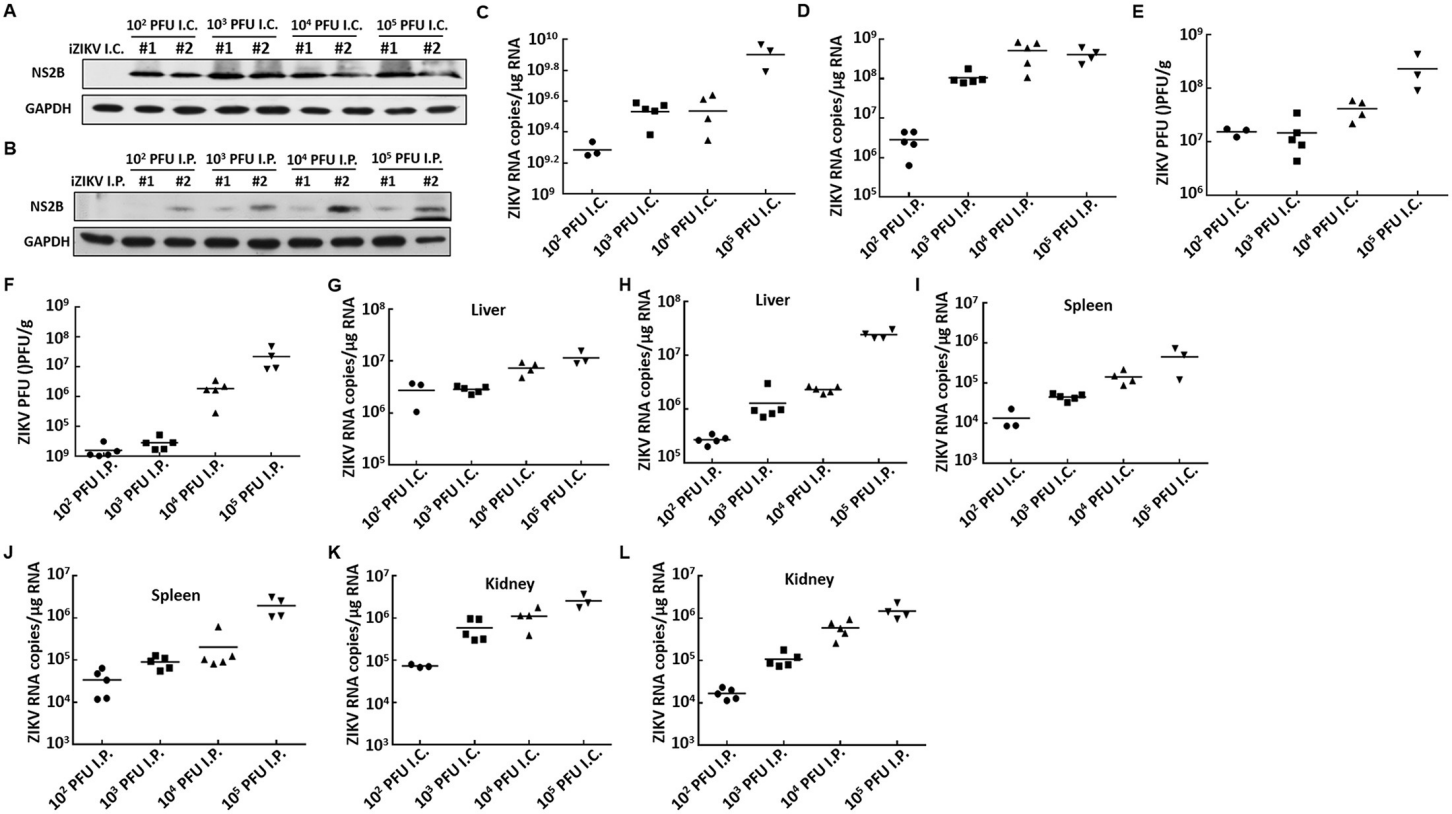
ZIKV replication in different organs of mice that underwent i.p. and i.c. injection of various doses of ZIKV. Six-day-old ICR suckling mice received 10^2^ to 10^5^ PFU ZIKV by i.c. or i.p. injection. Mice were sacrificed, and the brain, liver, spleen, and kidney tissues were collected to analyze ZIKV RNA replication and protein synthesis. (A and B) ZIKV protein synthesis in the brain tissues of mice that received ZIKV by (A) i.c. or (B) i.p. as detected by Western blot analysis with anti-ZIKV NS2B antibody. GAPDH served as equal loading control. (C and D) ZIKV RNA levels in the brain tissue of mice that received ZIKV by (C) i.c. or (D) i.p. as determined by qRT-PCR analysis. The ZIKV RNA level was normalized by the cellular *gapdh* mRNA level. (E and F) ZIKV viral titer in the brain tissue of mice that received ZIKV by (E) i.c. or (F) i.p. as determined by plaque forming assay. (G and H) ZIKV RNA levels in the liver tissue of mice that received ZIKV by (G) i.c. or (H) i.p. as determined by qRT-PCR analysis. (I and J) ZIKV RNA levels in the spleen tissue of mice that received ZIKV by (I) i.c. or (J) i.p. as determined by qRT-PCR analysis. (K and L) ZIKV RNA levels in the kidney tissue of mice that received ZIKV by (K) i.c. or (L) i.p. as determined by qRT-PCR analysis.

The ZIKV infection caused neurological syndromes [20, 22]. Brain tissues were collected from mice that received i.c. (n = 3 to 5) and i.p. (n = 4 to 5) injection of 10^2^ to 10^5^ PFU ZIKV to determine whether the virus can replicate in the brain of ICR suckling mice. The brain tissues were homogenized, and ZIKV protein expression was analyzed by Western blot assay. As shown in Fig 2A, the ZIKV protein was expressed in the i.c. injection group (10^2^ to 10^5^ PFU), but the expression level was lower in the i.p. injection group (Fig 2B). We conducted absolute quantification real-time PCR to detect ZIKV RNA replication level in the suckling mouse brain. As shown in Fig 2C and 2D, the ZIKV RNA replication level ranged from 10^9^ to 10^10^ and from 10^6^ to 10^9^ copies/μg RNA in the i.c. and i.p injection groups, respectively. In addition, the ZIKV viral load in the ICR suckling mouse brain was determined. Based on the plaque assay, the ZIKV titer in the i.c. group (from 10^7^ to 10^9^ PFU/g tissue) was approximately 2 to 3 log higher than that in the i.p. group (from 10^4^ to 10^8^ PFU/g tissue) injected with different concentrations of ZIKV (Fig 2E and 2F). To further characterize ZIKV replication in the suckling mice, we collected, weighed, and homogenized liver, spleen, and kidney tissues. The absolute quantification real-time PCR analysis showed that, regardless of organs or concentrations of ZIKV injected, ZIKV RNA was detected. The RNA replication level ranged from 10^5^ to 10^8^ copies/μg RNA in the liver, from 10^4^ to 10^7^ copies/μg RNA in the spleen, and from 10^5^ to 10^7^ copies/μg RNA in the kidney (Fig 2G–2L).

### ZIKV induced monocyte infiltrated into the brain of ICR suckling mice

**Fig 3:**
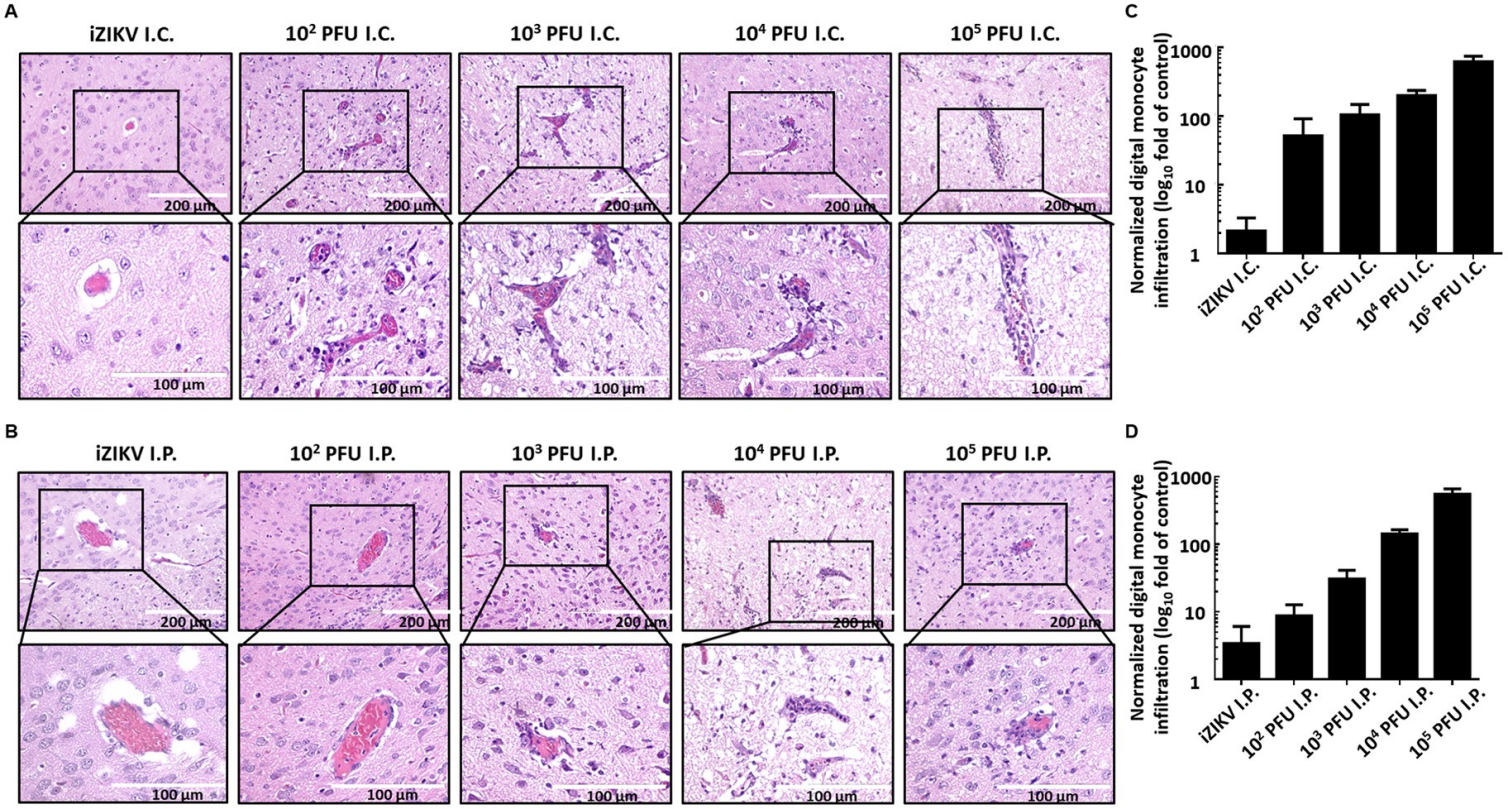
Monocyte infiltration in the brain of ICR suckling mice that received i.c. injection of various doses of ZIKV. Six-day-old ICR suckling mice received 10^2^ to 10^5^ PFU ZIKV by i.c. or i.p. injection. Mice were sacrificed, and the brain tissues of mice that received ZIKV by (A) i.c. or (B) i.p. were collected and subjected to hematoxylin and eosin staining. The biopsy specimens were examined by microscope. (C, D) The quantitation of monocyte infiltration by IHC assay.

The immune cell infiltration to the brain leads to inflammation, which is associated with ZIKV-caused GBS and other neurological disorders [21]. To investigate whether ZIKV infection can induce monocyte infiltration in the brain of ICR suckling mice, we collected brain tissues, stained them with hematoxylin and eosin (H&E staining), and observed under a photomicroscope. As shown in Fig 3A, the monocyte infiltration in the surrounding adjacent blood vessel in the brain of mice in the ZIKV i.c. injection group gradually increased compared with that in the mock infection group. As predicted, monocyte infiltration in the surrounding adjacent blood vessel was also observed in the brain of mice in the ZIKV i.p. injection group (Fig 3B). We further performed an immunohistochemistry (IHC) assay using specific monocytic marker Ly6C to quantify monocyte infiltration (Fig 3C and 3D) from the stained image (S1 Fig). Hence, the ICR suckling mouse model can be an immunocompetent model for understanding ZIKV pathogenesis.

### ZIKV invaded brain tissue of ICR suckling mice

**Fig 4:**
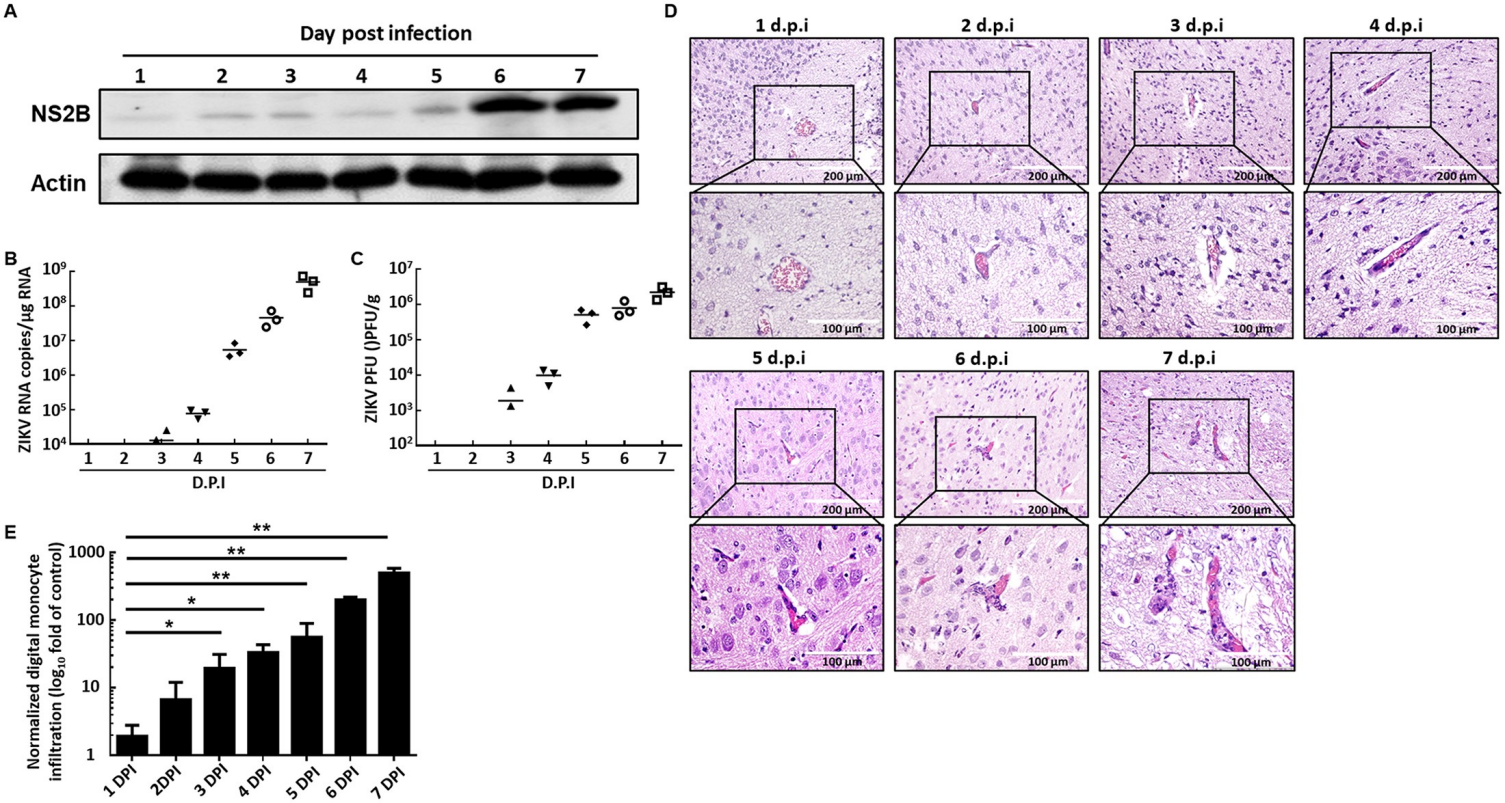
ZIKV replication in the brain of mice i.p. injected with 10^4^ PFU ZIKV. Six-day-old ICR suckling mice received 10^4^ PFU ZIKV by i.p. injection, and brain tissues were collected every day (n = 3) for analysis of ZIKV (A) protein synthesis, (B) RNA replication, (C) viral titer, and (D) monocyte infiltration. (E) The quantitation of monocyte infiltration by IHC assay. *P < 0.05; **P<0.01.

To further characterize the invasion kinetics of ZIKV in mouse brain, we injected 6-day-old ICR suckling mice through i.p. with 10^4^ PFU ZIKV and collected brain tissues (n = 3) for analyses of ZIKV protein synthesis, RNA replication, viral titration, and monocyte infiltration. ZIKV protein synthesis and RNA replication were analyzed through Western blot and absolute quantification real-time PCR analyses, respectively. Our results showed that the ZIKV protein synthesis gradually increased in the brain tissue and dramatically increased at 6 dpi (Fig 4A). The absolute quantification real-time PCR results also showed that ZIKV RNA replication gradually increased in the brain tissue and dramatically increased at 5 dpi (Fig 4B). As predicted, the ZIKV viral titer also increased in the brain tissue in a time-dependent manner (Fig 4C). These data indicated that ZIKV injected by i.p. can pass through the blood–brain barrier and replicate in the brain tissue. To further investigate the monocyte infiltration kinetics in the brain of ZIKV-infected ICR suckling mice, we first observed the monocyte infiltration by a photomicroscope following H&E staining (Fig 4D). Then, we performed an IHC assay to quantify the monocyte infiltration (Fig 4E and S2 Fig). Collectively, monocyte infiltration was observed at approximately the same time when ZIKV was detected in the brain tissue.

### Azithromycin prevented ZIKV-induced lethality and inhibited ZIKV replication in the suckling mouse model

**Fig 5:**
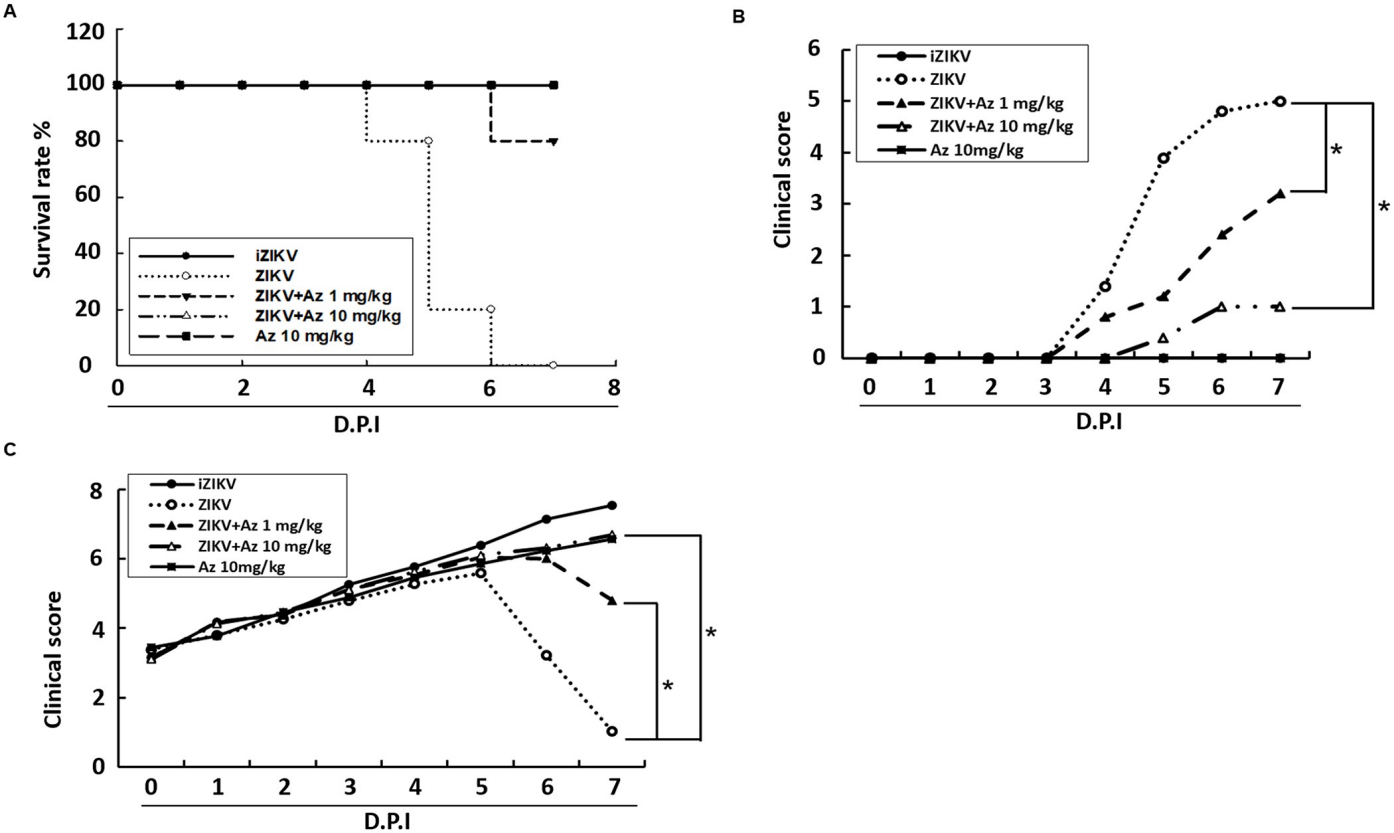
Survival rate, body weight, and clinical score in ZIKV-infected ICR suckling mice treated with Az. Six-day-old ICR suckling mice were divided into four groups. Group 1 received heat inactivated ZIKV; group 2 received 10 mg/kg of Az but without ZIKV infection (Az 10 mg/kg); group 3 received activated ZIKV and saline treatment; group 4 received activated ZIKV and 1 mg/kg Az; and group 5 received activated ZIKV and 10 mg/kg Az. (A) Survival rate, (B) clinical score, and (C) body weight were recorded every day. Mice were sacrificed at 7 dpi. Disease severity was scored as follows: 0: healthy, 1: body weight loss and ruffled fur, 2: lethargy and unsteady gait, 3: kinetic tremors and severe ataxia, 4: paralysis, and 5: death. *P < 0.05.

**Fig 6:**
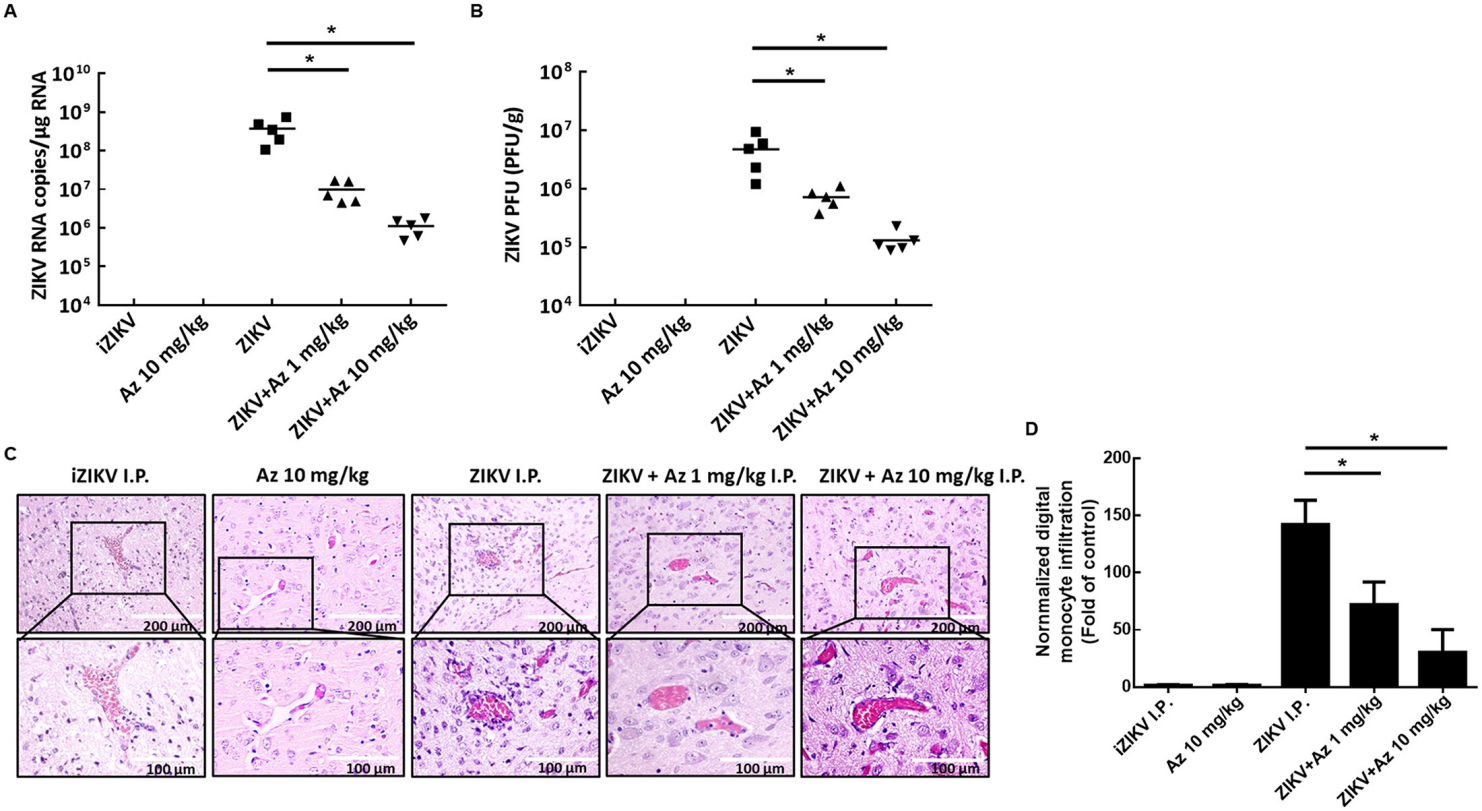
ZIKV replication and ZIKV-induced monocyte infiltration were suppressed by Az treatment in ZIKV-infected ICR suckling mice. Six-day-old ICR suckling mice were divided into four groups. Group 1 received heat inactivated ZIKV; group 2 received 10 mg/kg of Az but without ZIKV infection (Az 10 mg/kg); group 3 received activated ZIKV and saline treatment; group 4 received activated ZIKV and 1 mg/kg Az; and group 5 received activated ZIKV and 10 mg/kg Az. Mice were sacrificed at 7 dpi, and the brain tissues were collected for analysis of (A) ZIKV RNA replication, (B) viral titer, and (C) monocyte infiltration. (D) The quantitation of monocyte infiltration. *P < 0.05.

To evaluate whether ICR suckling mouse model is suitable for screening drugs for anti-ZIKV and ZIKV-induced disease, we used the well-characterize anti-ZIKV compound, namely, azithromycin (Az) [23, 24]. Six-day-old ICR suckling mice weighing 3.5–4 g were randomly divided into four groups. The mice were i.p. injected with ZIKV and Az (1 or 10 mg/kg) at 1, 3, and 5 dpi. Survival rate, clinical score, and body weights of ZIKV-injected mice treated with or without Az were measured daily for 7 days. Mice inoculated with heat-inactivated ZIKV (iZIKV) or 10 mg/kg of Az (Az 10 mg/kg) were used as mock controls. All the mice were sacrificed at 7 dpi, and their brain tissues were collected for analysis of ZIKV protein synthesis, RNA replication, viral titration, and monocyte infiltration. As shown in Fig 5A, ZIKV-infected mice that were not treated with Az developed severe sickness, leading to death within 4–7 dpi, in contrast to iZIKV-infected control mice. Moreover, Az administered at 1 and 10 mg/kg shielded 80% and 100% of mice from the life-threatening ZIKV infection compared with non-Az-treated mice. After 7 days of observation, the ZIKV-infected group showed signs of illness and neurological disease and exhibited 75% body weight loss compared with the iZIKV group (Fig 5B and 5C). The Az-administered group showed slight signs of illness and neurological disease as well as 50% (1 mg/kg) and 15% (10 mg/kg) body weight loss than the iZIKV group (Fig 5B and 5C). Moreover, injection of 1 mg/kg Az decreased the ZIKV RNA copies and viral titer by 1.58±0.27 and 0.81±0.18 log_10_, respectively. Meanwhile, injection of 10 mg/kg Az decreased the ZIKV RNA copies and viral titer by 2.52±0.25 and 1.55±0.16, respectively, compared with that in ZIKV-infected mice that were not treated with Az (Fig 6A and 6B). As predicted, Az treatment decreased the monocyte infiltration into the brain of ZIKV-infected ICR suckling mice in a dose-dependent manner (Fig 6C and 6D). In addition, mice received 10 mg/kg of Az exhibited no any side effect. These data indicated that ICR suckling mouse model is suitable for screening drugs against ZIKV and ZIKV-induced diseases.

## Discussion

In the present study, we established a ZIKV-infected ICR suckling mice model for investigating the propagation of ZIKV infection and, especially, the antiviral drug development *in vivo*. The ICR suckling mice model is susceptible for ZIKV infection by using both i.p. and i.c. injection methods and provide infection with high viral loads in the brain, which is consistent with severe neurological symptom in human [12, 14, 25]. Our results further clearly illustrated the level of ZIKV propagation in different tissues, including liver, spleen, and kidney, which indicated that ZIKV distributed systemically in the ICR suckling mice and provided the evidence of ZIKV distribution in the infected individuals. Recently, Retallack et al. reported that Az could prevent ZIKV infection *in vitro* [23]. Consistently, our study further confirmed that Az could protect mice from lethal ZIKV infection *in vivo*. To date, ZIKV has been already spread out of the world for years, therefore, a therapeutic treatment against ZIKV infection is urgently needed. Retallack et al. and our results provide a potential therapeutics for treatment of ZIKV infection.

Recently, most animal models for investigating ZIKV mainly focus on the ZIKV-caused neurological injury and disruption of neural development [26–28]. For instance, Oh et al. revealed that ZIKV not only infected central nervous system but also the peripheral neurons in mouse embryo, and their study demonstrated that ZIKV infection activated cell apoptosis pathway in neuron cells [26]. Li et al. further delineated the comprehensive picture of pathogenesis of ZIKV and microcephaly in mouse embryo [27], in which they demonstrated that ZIKV infected neural progenitor cells (NPCs) to cause cell-cycle arrest, leading to the defective differentiation of NPCs, which resulted in apoptosis of post-mitotic neurons for generation of microcephaly through induction of immune response in brain. In addition, Duggal et al. [29] and Li et al. [28] have established mice model for studying ZIKV infection. However, their reports provided less evidence of the ZIKV-caused monocyte infiltration and did not provide the detail protocol for evaluation of anti-ZIKV agent. In the present study, we provided the evidence of ZIKV distribution in the organs. In addition, we detailedly determined the virus load in different organs and the monocyte infiltration in the suckling mice model by ZIKV infection with different routes and doses. Importantly, we established a fast and easy-performing animal model for evaluation of anti-ZIKV agent *in vivo*.

Animal models for understanding the transmission and infection of ZIKV has been developed in the different strains of mice, such as AG129, C57BL/6, and Irf3/5/7 triple knockout (TKO) mice [20]. AG129 and Irf3/5/7 TKO mice are the immunocompromised mice which were permissive to different strain ZIKV infection and vulnerable to ZIKV-relative pathology and lethality [10, 21, 30, 31]. Similarly, the ICR suckling mice model allowed the investigation of some aspect of ZIKV-caused symptom including viral-relative mortality, clinical score increasing, and viral propagation rising [22, 32]. Furthermore, the immune cell infiltration was observed in the ZIKV-infected ICR suckling mice model. There observations in ICR suckling mice may provide the phenomenon for investigation of the efficacy of developing drug or vaccine, and the tissue of the suckling mice may use to underlying the mechanism discovery [20]. However, the limitation of AG129 mice is unable to develop the vaccine or drug that is dependent on the intact interferon pathway [33]. Furthermore, AG129 and Irf3/5/7 TKO mice has been indicated to be uncommonly available in some research laboratories [33, 34]. The ICR suckling mice model are the immunocompetent mice, which have allowed researchers to investigate the interferon-dependent antiviral compounds or vaccine [20, 35]. Furthermore, the suckling mice model is much more common and easy to establish in the most research laboratories [36]. However, the age of the suckling mice lead draws some limitations that the viremia in the blood was hard to collect and proceed, and the study of vertical transmission was not available. Collectively, we developed the ZIKV-infected ICR suckling mice model with a productive viral replication accompanied with some clinical manifestation, including symptom kinetic and immune cells infiltration in the brain. The model thus may provide protocols and information for researchers to investigate antiviral effect or the mechanism determination in a time-saving method and easy operation.

## Materials and methods

### Ethics statement and experimental animals

Six-day-old ICR suckling mice weighing 3.5–4 g and ICR strain breeder mice were purchased from BioLasco Taiwan Co. Ltd (Taipei City, Taiwan). All animal experiments were performed under specific pathogen-free conditions. The experimental methods were carried out according to the Guide for the Care and Use of Laboratory Animals. The experimental procedures were approved by the Animal Research Committee of Kaohsiung Medical University of Taiwan (IACUC, 105198) under the guidance of the Public Health Service Policy on Humane Care and Use of Laboratory Animals. All mice received humane care and were given standard diet and water ad libitum. Prior to the experiment, mice were acclimatized for a week under the standard laboratory condition following the Animal Use Protocol of Kaohsiung Medical University.

### Evaluation of susceptibility of ICR suckling mice to ZIKV infection

ZIKV strain MR-766 was amplified in C6/36 mosquito cells and titrated in Vero cells. Six-day-old ICR suckling mice were inoculated with 10^2^ (10^2), 10^3^ (10^3), 10^4^ (10^4), and 10^5^ (10^5) PFU ZIKV by i.p. and i.c. injection. Mice injected with heat inactivated ZIKV (iZIKV) were used as mock control. Survival rate, body weight, and clinical score were measured every day after ZIKV infection. The clinical scores were recorded according to illness symptoms: 0: healthy, 1: body weight loss and ruffled fur, 2: lethargy and unsteady gait, 3: kinetic tremors and severe ataxia, 4: paralysis, and 5: death. The clinical score 5 indicates death, which includes the dead and euthanasia. The criterion for euthanasia is that the mice exhibited no movement, uncontrollable behavior, spastic movements, or do not return to upright position if put on its side. The body weight of the euthanized mice is included in the calculation of body weight, in contrast, the body weight of dead mice is excluded in the calculation of body weight [37–42]. The mice were sacrificed by CO_2_ asphyxiation at 8 dpi. In brief, 0.1g of brain, liver, spleen, and kidney tissues were harvested by R.I.P.A buffer, TRIzol reagent, and RPMI medium for protein, RNA, and virus collection, respectively.

### Western blot assay

Western blot assay was performed as described previously [37, 43]. In brief, protein from cell lysates was separated by SDS-PAGE and transferred onto PVDF membrane. Signal was detected using an ECL detection kit (PerkinElmer, CT). Antibodies used in the study included anti-ZIKV NS2B antibody (1:3,000; GeneTex) and anti-actin antibody (1:10,000; GeneTex).

### Quantitation of viral RNA copy number

Total cell RNA was extracted by TRIzol^™^ reagent following the manufacturer’s protocol. Both double- and single-stranded DNA in the RNA samples were denatured by using RQ1 (RNA Qualified) RNase-Free DNase (Promega). The RNA samples were reverse transcribed into complementary DNA by using the M-MLV Reverse Transcription System (Promega). Absolute quantitation of ZIKV RNA copies was conducted relative to the standard curves. RT-qPCR analysis was conducted using serially diluted expression plasmids containing the coding sequence of ZIKV NS5 with the following specific primers: forward primer, 5′-aagcaaaaggtagccgcgcc-3′, and reverse primer, 5′-tgtcccagccagcagtgtca-3′, targeting the ZIKV NS5 gene. The reactions were performed using ABI Step One real-time PCR-system (ABI Warrington, UK).

### Plaque assay

Vero cells were seeded in 24-well plates at 9 × 10^4^ per well. Virus collected from mouse brain was serially diluted and incubated with Vero cells to a volume of 200 μL at 37 °C. After 2h of incubation, the medium was refreshed with MEM containing 2% FBS and 0.8% methyl cellulose (Sigma–Aldrich). At 3 dpi, the cells were fixed with 4% paraformaldehyde for 15 min and stained with the crystal violet solution (1% crystal violet and 0.64% NaCl) at 25°C for 1h. Viral titer was calculated by observation of plaque formation.

### Histopathology

Histopathological observation was performed as previously described [44]. In brief, each tissue was harvested and subjected to H&E or IHC staining to observe tissue injury or monocyte infiltration under a photomicroscope.

### Quantitation of monocyte infiltration

To quantify the monocyte infiltration in brain tissues, the slices of brain tissues were first received immunohistochemistry (IHC) staining with anti-Ly6C antibody (Abcam), and then the degree of monocytes infiltration was digitally quantified by ImageJ software [45–47]. In brief, the staining patterns of Ly6C were detected by EVOS FL Cell Imaging System (Thermo Fisher Scientific), and the blot signals from the digital images were further analyzed by Image J software (NIH, USA). The results were normalized by the scanning area and were presented as the log_10_ fold-change compared to that of uninfected (iZIKV) control defined as 1.

### Reagents

Az was obtained from Sigma (St. Louis, MO, USA).

### Evaluation of brain kinetics of ICR suckling mice to ZIKV infection

Six-day-old ICR suckling mice were i.p. injected with 10^4^ PFU ZIKV. Three mice were randomly chosen to be sacrificed by CO_2_ asphyxiation, and brain tissues were collected each day. All mice were sacrificed at 7 dpi. The brain tissues were collected by R.I.P.A buffer, TRIzol reagent, and RPMI medium for protein, RNA, and virus collection, respectively.

### Evaluation of anti-ZIKV replication activity assay in ICR suckling mouse model

Six-day-old ICR suckling mice weighing 3.5–4 g were randomly divided into four groups: group 1: i.p. injectedwith 10^4^ PFU heat-inactivated ZIKV (iZIKV); group 2 received 10 mg/kg of Az but without ZIKV infection (Az 10 mg/kg); group 3: i.p. injected with 10^4^ PFU ZIKV and saline (ZIKV); group 4: i.p. injected with 10^4^ PFU ZIKV and 1 mg/kg Az (ZIKV+Az 1mg/kg); and group 5: i.p. injected with 10^4^ PFU ZIKV virus and 10mg/kg Az (ZIKV+Az 10mg/kg). The ZIKV-infected mice were i.p. injected with the tested agents at 1, 3, and 5 dpi. Survival rate, body weight, and clinical score were recorded every day post ZIKV infection. The clinical score was recorded according to the illness symptoms: 0: healthy, 1: body weight loss and ruffled fur, 2: lethargy and unsteady gait, 3: kinetic tremors and severe ataxia, 4: paralysis, and 5: death. The mice were sacrificed by CO_2_ asphyxiation at 7 dpi. The brain tissues were collected by R.I.P.A buffer, TRIzol reagent, and RPMI medium for protein, RNA, and virus collection, respectively.

### Statistical analysis

Data were expressed as mean ± SD of at least three independent experiments. Statistical calculations were analyzed by Student’s t-test using GraphPad Prism 6 (GraphPad Software Inc.).

## Supporting information

S1 Fig ZIKV infection increases monocyte infiltration in mice brain tissues in a dose-dependent manner. (A–B) Six-day-old ICR suckling mice received 10^2^ to 10^5^ PFU ZIKV or heat inactivated ZIKV (iZIKV) by i.c. or i.p. injection. The brain tissues of mice received ZIKV by (A) i.c. or (B) i.p. were collected and subjected to immunohistochemistry staining with anti-Ly6C antibody.
(TIF)

S2 Fig ZIKV infection increases monocyte infiltration in mice brain in a time-dependent manner. Six-day-old ICR suckling mice received 10^4^ PFU ZIKV by i.p. injection, and brain tissues were collected every day (n = 3) for analysis of ZIKV monocyte infiltration by IHC staining with anti-Ly6C antibody.
(TIF)

S3 Fig ZIKV-induced monocyte infiltration are suppressed by Az treatment in ICR suckling mice. Six-day-old ICR suckling mice were divided into four groups. Group 1 received heat inactivated ZIKV; group 2 received 10 mg/kg of Az but without ZIKV infection (Az 10 mg/kg); group 3 received activated ZIKV and saline treatment; group 4 received activated ZIKV and 1 mg/kg Az; and group 5 received activated ZIKV and 10 mg/kg Az. Mice were sacrificed at 7 dpi, and the brain tissues were collected for analysis of monocyte infiltration by IHC staining with anti-Ly6C antibody.
(TIF)
