# Supplementary figures and images for: ICR suckling mouse model of Zika virus infection for disease modeling and drug validation
Source: PLoS Negl Trop Dis. 2018 Oct 24;12(10):e0006848. doi: 10.1371/journal.pntd.0006848 (PMC6218097; doi:10.1371/journal.pntd.0006848)

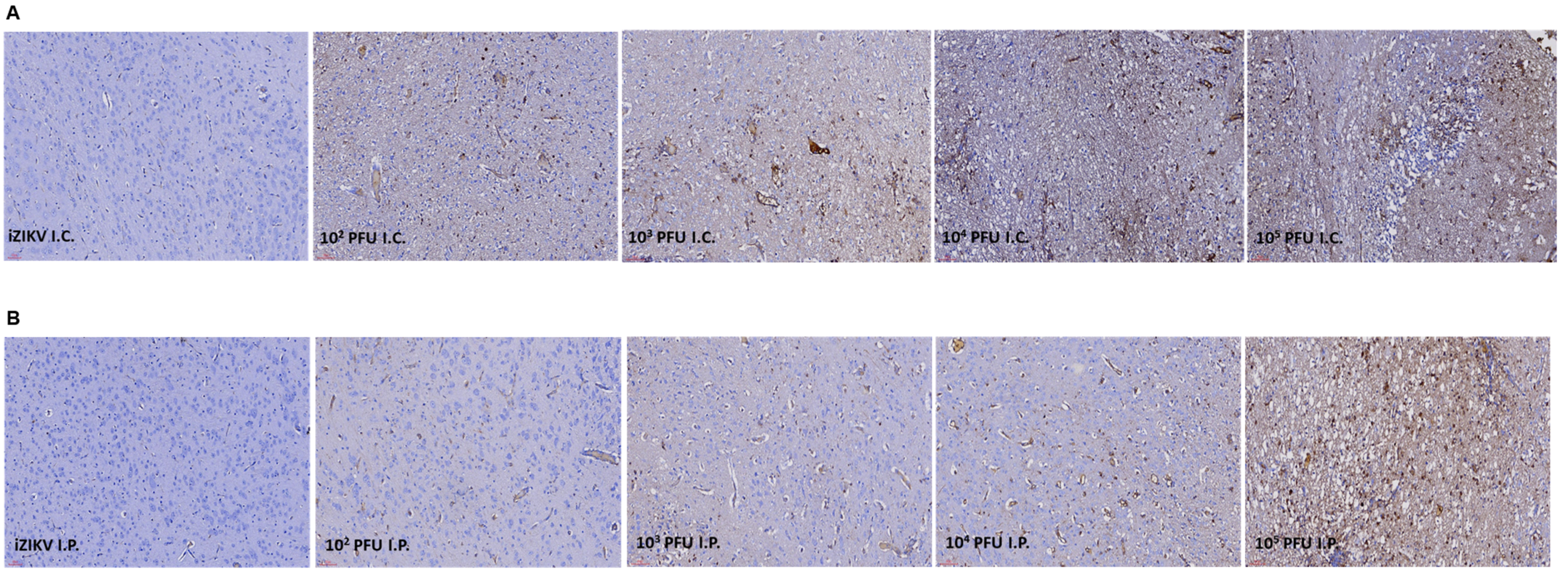

Supplement: S1 Fig — (A–B) Six-day-old ICR suckling mice received 102 to 105 PFU ZIKV or heat inactivated ZIKV (iZIKV) by i.c. or i.p. injection. The brain tissues of mice received ZIKV by (A) i.c. or (B) i.p. were collected and subjected to immunohistochemistry staining with anti-Ly6C antibody. (TIF) [file pntd.0006848.s001.tif]

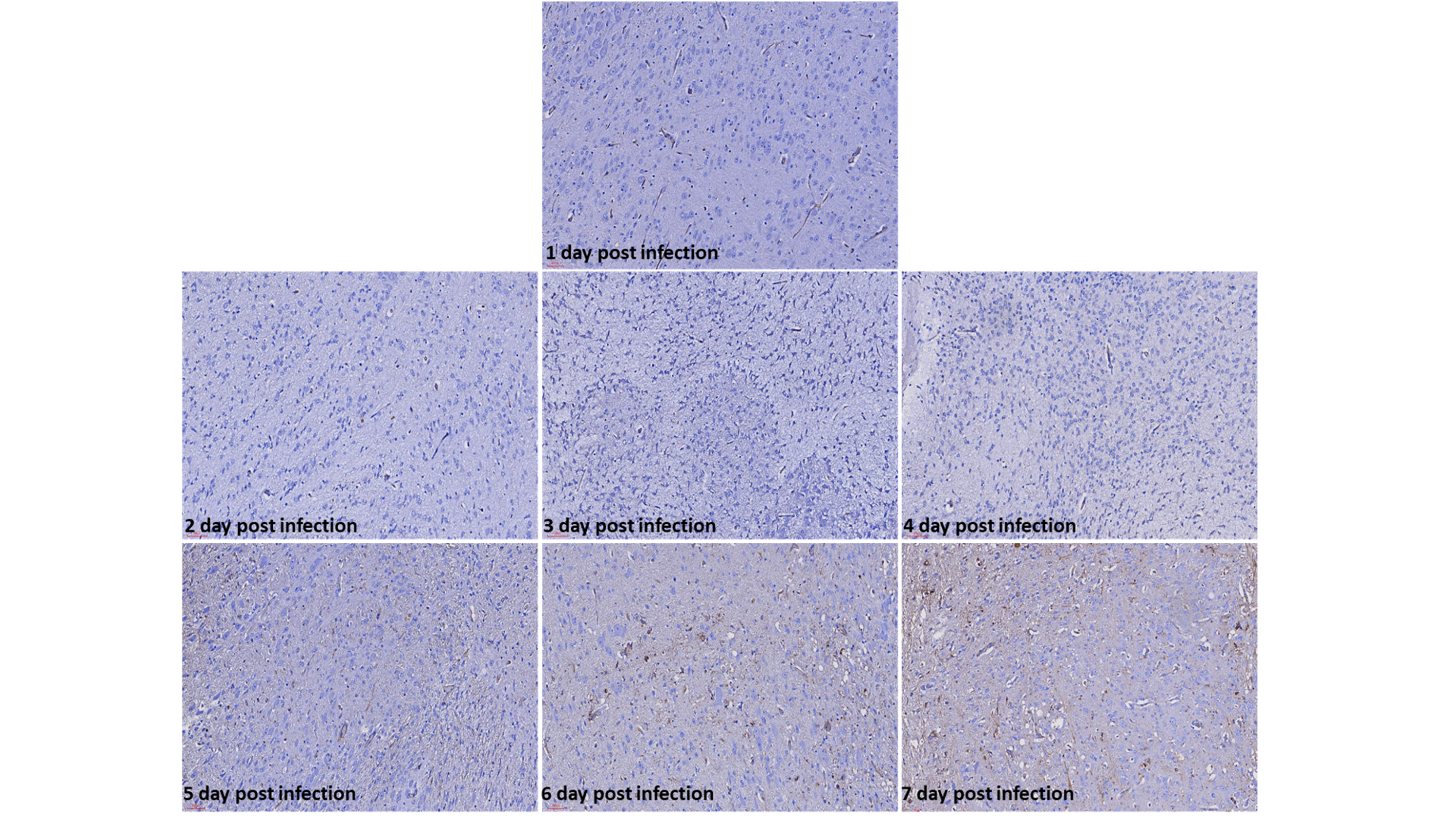

Supplement: S2 Fig — Six-day-old ICR suckling mice received 104 PFU ZIKV by i.p. injection, and brain tissues were collected every day (n = 3) for analysis of ZIKV monocyte infiltration by IHC staining with anti-Ly6C antibody. (TIF) [file pntd.0006848.s002.tif]

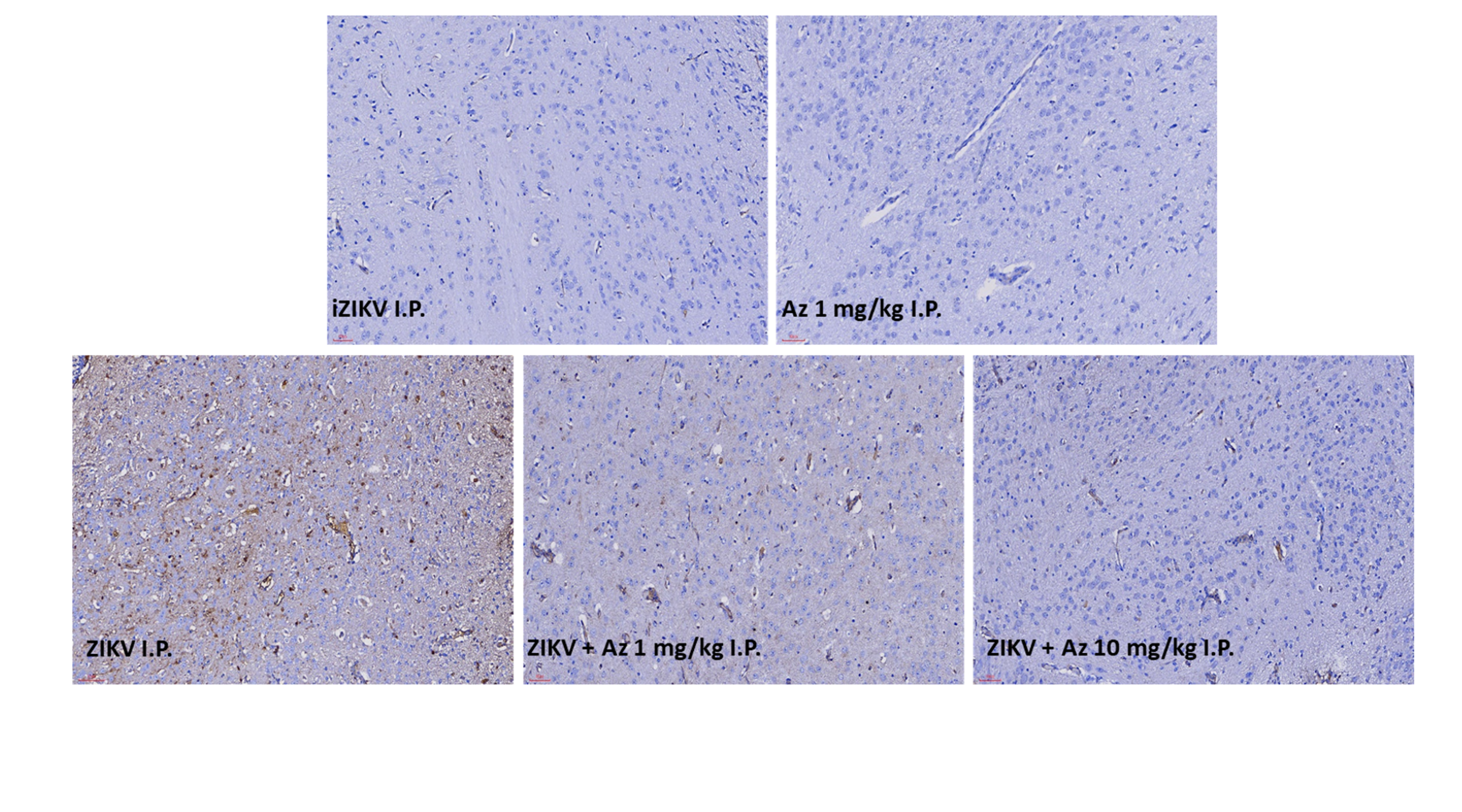

Supplement: S3 Fig — Six-day-old ICR suckling mice were divided into four groups. Group 1 received heat inactivated ZIKV; group 2 received 10 mg/kg of Az but without ZIKV infection (Az 10 mg/kg); group 3 received activated ZIKV and saline treatment; group 4 received activated ZIKV and 1 mg/kg Az; and group 5 received activated ZIKV and 10 mg/kg Az. Mice were sacrificed at 7 dpi, and the brain tissues were collected for analysis of monocyte infiltration by IHC staining with anti-Ly6C antibody. (TIF) [file pntd.0006848.s003.tif]
